# Supplementary material for: Pyrroloquinoline Quinone Alleviates Intestinal Inflammation and Cell Apoptosis via the MKK3/6-P38 Pathway in a Piglet Model
Source: Int J Mol Sci. 2024 Sep 8;25(17):9723. doi: 10.3390/ijms25179723 (PMC11395797; doi:10.3390/ijms25179723)
Supplement: Supplementary file 1 [file ijms-25-09723-s001.zip › ijms-3034666-supplementary.pdf]

Supplemental Table S1 Ingredient composition and nutrient levels of the experimental diets (%, as-fed basis)

**Ingredients (%)**	**CTRL**	**PQQ**	**Nutrient levels**	
Corn	54.74	54.44	Analytical value	
Soybean meal	14.50	14.50	Crude protein (%)	18.70
Extruded full-fat soybean	7.00	7.00	Calcium (%)	0.73
Soy protein concentrate	7.00	7.00	Calculated value	
Fish meal	5.50	5.50	DE (Mcal/kg)	3.50
High protein whey powder	4.00	4.00	Digestible phosphorus (%)	0.43
Sucrose	2.00	2.00	Lys (%)	1.32
Soybean oil	2.00	2.00	Met + cys (%)	0.73
Limestone	0.80	0.80	Thr (%)	0.82
Dicalcium phosphate	0.80	0.80	Trp (%)	0.22
Salt	0.25	0.25		
L-Lysine·HCL (78.8%)	0.48	0.48		
Threonine (98.5%)	0.16	0.16		
Tryptophan (98.5%)	0.02	0.02		
Methionine (99.0%)	0.25	0.25		
Pyrroloquinoline quinone	0.00	0.30		
Premix (free-antibiotics)[1]	0.50	0.50		

[1]Premix provided the following per kg: Vitamin A, 10,000 IU; Vitamin D3, 2,300 IU; Vitamin E, 25 IU; Vitamin K3, 2.40 mg; Thiamin, 2.0 mg; Riboflavin, 4.0 mg; Pyridoxine, 3.0 mg; Vitamin B12, 12 μg; Niacin, 30 mg; Pantothenic acid, 13 mg; Folic acid, 1 mg; Biotin, 50 μg; Fe, 9 mg; Cu, 20 mg; Zn, 90 mg; Mn, 20 mg; I, 0.35 mg; Se, 0.3 mg.

Supplemental Table S2 Information of primary antibodies used for western blotting analysis

Protein	Catalog no.	Source	Company
p38	#8690	Rat	ProteinTech Group
ZO-1	#5406	Rat	Cell Signaling Technology
ZO-2	#2874	Rat	Cell Signaling Technology
ZO-3	ab205882	Rat	Abcam
Occludin	27260-1-AP	Rat	ProteinTech Group
Claudin-1	13050-1-AP	Rat	ProteinTech Group
p-p38	#4511	Rat	Cell Signaling Technology
MKK3/6	70R-51621	Rat	Fitzgerald
PERK	24390-1-AP	Rat	ProteinTech Group
Bax	50599-2-lg	Rat	ProteinTech Group
Bcl-2	12789-1-AP	Rat	ProteinTech Group
Caspase-3	16977-1-AP	Rat	ProteinTech Group
Tubulin	#2125	Rat	Cell Signaling Technology
β-actin	#4970	Rat	Cell Signaling Technology

Supplemental Table S3 Differentially upregulated proteins in jejunal mucosa

Uniprot KB no.	Gene name	Protein name	log_2FC
A0A287ABR2	NCOA4	Nuclear receptor coactivator 4	1.36
I3LU89	CHMP1A	Charged multivesicular body protein 1a	1.36
F1S3U0	GALT	Galactose-1-phosphate uridylyltransferase	1.39
F1S1G8	LOC110256000	Amine oxidase	1.43
F1RVP4	MOCS1	Cyclic pyranopterin monophosphate synthase	1.47
A0A286ZJK2	HNRNPM	Heterogeneous nuclear ribonucleoprotein M	1.49
A0A287B1A2	UTP4	UTP4 small subunit processome component	1.50
A1YH88	SLA-6	MHC class I antigen 6	1.53
F1SP08	PTPN3	Tyrosine-protein phosphatase non-receptor type	1.54
A0A286ZJG3	C6orf222	Bcl-2 interacting protein 5	1.54
F1RSJ2	TRAPPC9	Uncharacterized protein	1.54
F1SS24	FN1	Fibronectin	1.59
L8B139	IGHG	IgG heavy chain	1.64
F1S9X9	HSH2D	Hematopoietic SH2 domain containing	1.69
F1SH35	ZFC3H1	Zinc finger C3H1-type containing	1.69
F1S1U4	TLK1	Tousled like kinase 1	1.74
I3L9F7	KPNA1	Karyopherin subunit alpha 1	1.75
F1RPX2	EML3	EMAP like 3	1.76
A0A287B3U8	RTL4	Retrotransposon gag like 4	1.77
F1SUK6	CAPN5	Calpain 5	1.79
F1S6M7	unknow	Tubulin beta chain	1.81
A0A287A6Q3	ASH2L	ASH2 like, histone lysine methyltransferase complex subunit	1.81
F2Z4Z4	CSNK1A1	Casein kinase I isoform alpha	1.82
F1SUZ2	NUP98	Nuclear pore complex protein Nup96	1.82
F1SU73	ECD	Ecdysoneless cell cycle regulator	1.85
A0A287AC46	TNRC6B	Trinucleotide repeat containing adaptor 6B	1.91
F1S8G3	MRPS33	Mitochondrial ribosomal protein S33	1.91
Uniprot KB no.	Gene name	Protein name	log_2FC
I3LN42	GC	Gc-globulin	2.01

A0A287B205	LOC110255185	C-type lectin domain-containing protein	2.03
F1SD83	NACC1	Nucleus accumbens associated 1	2.09
K7GP80	EIF2AK3	Eukaryotic translation initiation factor 2 alpha kinase 3	2.11
A0A287AAP9	PSTK	Phosphoseryl-tRNA kinase	2.11
A0A286ZS99	RTN2	Reticulon	2.22
A0A286ZY95	FN1	Fibronectin	2.26
A0A286ZPX7	PYM1	PYM homolog 1, exon junction complex associated factor	2.32
F1SRN4	TRIO	Non-specific serine/threonine protein kinase	2.38
A0A287AHU4	CRNKL1	Crooked neck pre-mRNA splicing factor 1	2.38
A0A287AT98	TACC3	Transforming acidic coiled-coil containing protein 3	2.42
A0A287BRD8	BAG5	BCL2 associated athanogene 5	2.59
D3K5K8	MYST2	Histone acetyltransferase	2.60
F1SA26	ARG2	Arginase	2.62
A0A0B8RVY2	DHX8	Uncharacterized protein	2.67
F1RPY9	NAA40	N-alpha-acetyltransferase 40	2.86
F1SEG8	PLEKHG2	Pleckstrin homology and RhoGEF domain containing G2	2.90
A0A287BMI9	ARID4B	Uncharacterized protein	2.95
I3LM75	SPG7	Paraplegin	2.97
A0A0B8S0A5	CDK12	Cyclin-dependent kinase 12	2.99
I3LB53	LIN54	Lin-54 DREAM MuvB core complex component	3.14
F1SA41	MPP5	MAGUK p55 subfamily member 5	3.15
F1RG79	ZNF629	Zinc finger protein 629	3.21
A0A287BMW7	OGFOD3	2-oxoglutarate and iron dependent oxygenase domain containing 3	3.26
F1RX67	UBXN8	UBX domain protein 8	3.26
F1RUP8	CDC123	Cell division cycle protein 123 homolog	3.60
A0A287B8U5	FAM111A	Family with sequence similarity 111 member A	3.83
A0A287BLN7	PCSK5	P/Homo B domain-containing protein	4.48
A0A287A035	LOC102162880	Uncharacterized protein	4.52
A0A287AIU0	MFGE8	Lactadherin	5.05
A0A077ETG0	TRIM56	Tripartite motif containing 56	5.36

Q9N2H7	unknow	Poly-Ig receptor	5.79
F1RK85	MED15	Mediator of RNA polymerase II transcription subunit 15	5.82
F1S3T1	GUF1	Translation factor GUF1, mitochondrial	6.38
P08419	CELA2A	Chymotrypsin-like elastase family member 2A	6.53

Supplemental Table S4 Differentially down-regulated proteins in jejunal mucosa

Uniprot KB no.	Gene name	Protein name	log_2FC
F1RN44	LAMP1	Lysosomal associated membrane protein 1	-7.16
A0A287B3K6	RPL19	Ribosomal protein L19	-7.15
F1SV27	FHL3	Four and a half LIM domains 3	-5.15
A0A287BHU8	CACUL1	CDK2 associated cullin domain 1	-4.85
A0A2S1PUH7	SLA-2	MHC class I antigen 2	-4.65
A0A286ZSJ8	NT5C3A	5'-nucleotidase	-4.31
A0A286ZSX7	ULK3	Serine/threonine-protein kinase ULK3	-4.18
A0A287AFX0	DAB2IP	DAB2 interacting protein	-4.17
F1S9C2	GTF2H1	General transcription factor IIH subunit 1	-4.12
M3UZ68	GMEB1	Uncharacterized protein	-4.02
F1RFW5	RAB13	RAB13, member RAS oncogene family	-3.70
F1SUS9	MRPL48	39S ribosomal protein L48, mitochondrial	-3.46
Q52NJ5	ARL1	ADP-ribosylation factor-like protein	-3.37
Q52NJ5	ARL1	ADP-ribosylation factor-like protein	-3.37
A0A287BQ22	MGMT	6-O-methylguanine-DNA methyltransferase	-3.07
Q1HE49	AKR1C4	Uncharacterized protein	-3.07
I3LCZ8	CYP2C42	Cytochrome P450 2C42	-2.89
I3LKR2	TFF3	Trefoil factor 3	-2.78
A0A287B1Z9	BSCL2	Seipin	-2.77
A5A782	MYO7A	Myosin VIIA	-2.73
A0A287ALN3	MIEF1	Mitochondrial dynamics protein MID51 isoform X1	-2.64
F1SEH2	GMFG	Glia maturation factor	-2.62
C1IHT9	PDK4	Uncharacterized protein	-2.51
A0A287AS68	FCN1	Ficolin-1	-2.49
F1SNU2	ELP6	Elongator complex protein 6	-2.42
M3TYM9	DOCK8	Dedicator of cytokinesis 8 tv1	-2.40
F1RRY9	FAM102A	Uncharacterized protein	-2.37
A0A287B9I5	OPA3	Optic atrophy 3 protein isoform X1	-2.35
F1S7B0	HOOK1	Uncharacterized protein	-2.34
A0A287A743	LETMD1	LETM1 domain containing 1	-2.34

F1RUP0	ECHDC3	Enoyl-CoA hydratase domain containing 3	-2.33
A0A287AWH6	NDUFV3	Complex I-9kD	-2.28
I3LU36	USP25	Ubiquitin specific peptidase 25	-2.28
A0A287AWN1	MCRIP1	MAPK regulated corepressor interacting protein 1	-2.27
A0A286ZKT1	CAMK2G	Calcium/calmodulin-dependent protein kinase	-2.26
A0A287BED1	PFDN1	Prefoldin subunit 1	-2.24
F1S712	AIG1	Androgen induced 1	-2.24
A0A287AG82	PATJ	PATJ crumbs cell polarity complex component	-2.23
A0A287ATG8	NEK4	NIMA related kinase 4	-2.21
A0A286ZI26	PLGRKT	Plasminogen receptor with a C-terminal lysine	-2.20
C6K7I1	KPNA3	Importin subunit alpha	-2.18
B2D2K8	SLC3A1	Solute carrier family 3 member 1	-2.16
F1S8U4	MRPL43	39S ribosomal protein L43, mitochondrial isoform X2	-2.14
I3LG35	TRMT1L	TRMT1-like protein	-2.12
I3LA49	HERC5	HECT and RLD domain containing E3 ubiquitin protein ligase 5	-2.10
F1SJP1	TM4SF4	Transmembrane 4 L six family member 4	-2.01
F1RV28	MAP2K6	Mitogen-activated protein kinase kinase 6	-2.00
F1RNF1	LAIR1	Uncharacterized protein	-1.99
O19063	APCS	Serum amyloid P-component	-1.95
I3LI45	GIP	Gastric inhibitory polypeptide	-1.92
I3LAV9	TOX4	TOX high mobility group box family member 4	-1.91
A0A287AB58	ISG15	Ubiquitin-like modifier	-1.89
A0A286ZZ23	SCYL1	Protein kinase domain-containing protein	-1.85
F1SQD9	CRELD1	Protein disulfide-isomerase	-1.83
A0A287B441	ATP5PF	ATP synthase-coupling factor 6, mitochondrial	-1.81
P12026	DBI	Acyl-CoA-binding protein	-1.81
I3LQZ4	MARK3	Uncharacterized protein	-1.80
A0A287BFA5	RGL2	Uncharacterized protein	-1.79
A0A287AZP4	PPP4R2	Protein phosphatase 4 regulatory subunit 2	-1.78

F1SMV6	NCL	Nucleolin	-1.75
F1RSL0	EXOSC4	Exosome complex component RRP41	-1.74
A5A758	KRT1	Keratin 1 (Fragment)	-1.73
F1SER9	FAT1	Uncharacterized protein	-1.68
A0A287B207	UBE2R2	Ubiquitin conjugating enzyme E2 R2	-1.68
F1RIS6	MMAB	Corrinoid adenosyltransferase	-1.63
A0A287AHT1	PDK3	Protein-serine/threonine kinase	-1.62
Q28944	CTSL	Cathepsin L1	-1.61
C3VPJ4	CLDN7	Claudin	-1.59
F1SMU6	PIGN	GPI ethanolamine phosphate transferase 1	-1.59
I3LA25	SNRNP27	U4/U6.U5 small nuclear ribonucleoprotein 27 kDa protein	-1.57
A5A775	HPS6	Uncharacterized protein	-1.57
F1RGP5	SHC1	SHC-transforming protein 1	-1.46
A0A286ZJV0	SLC25A28	Mitoferrin-2	-1.45

Supplemental Figure S1 Data analysis and data quality evaluation results for

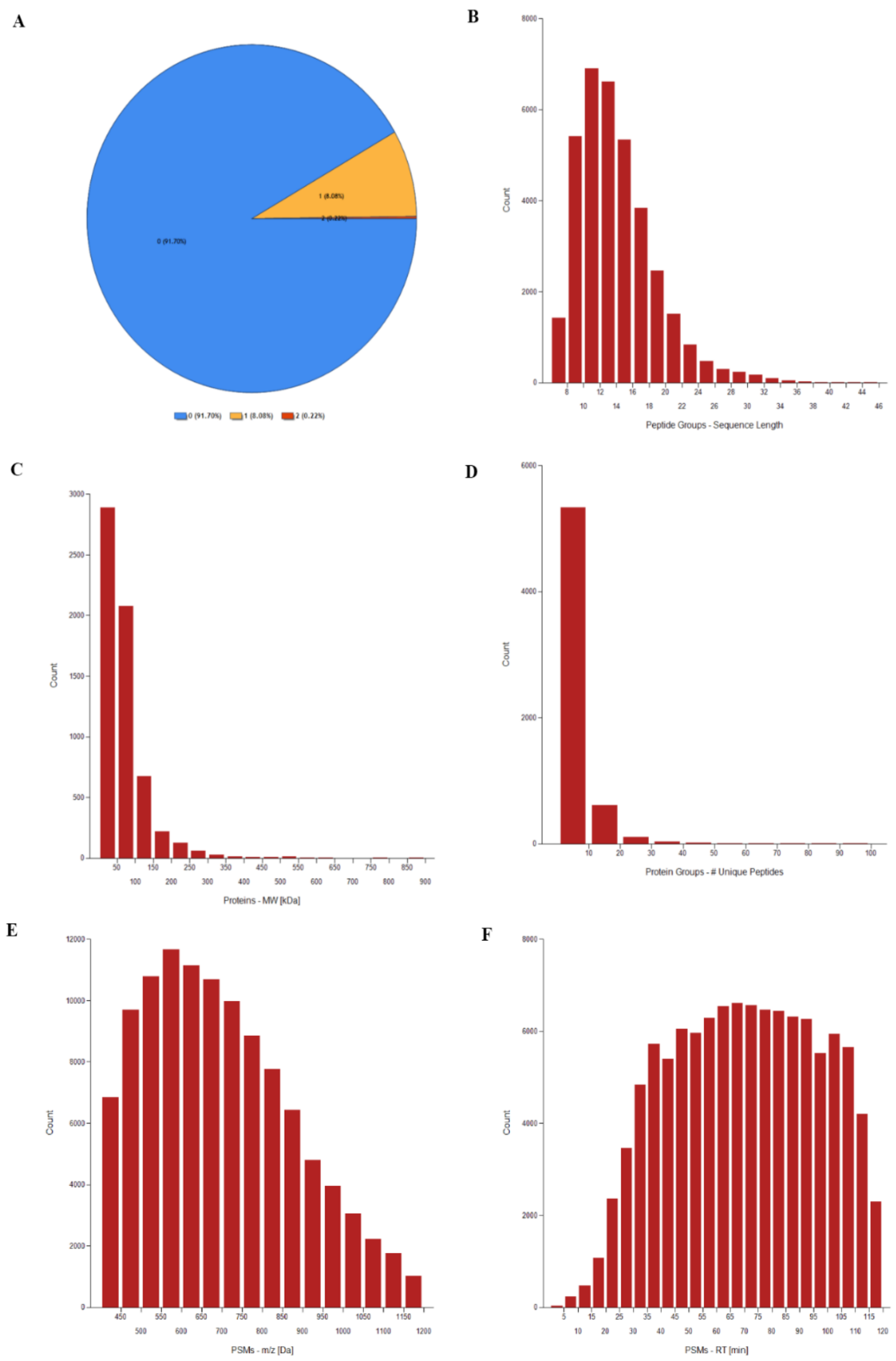

DIA

A, Distribution for leakage of peptide sites: each color denotes the leakage ratio of peptides sites; B, The interval and number representing amino acids length. C, The proteins molecular weight and number distribution in samples. D, Distribution of specific peptides. E, Relation between the number of peptides and m/z. F, Relation diagram between number of peptides and retention time.
